# Concern about the possibility of being a victim of extortion and symptoms of anxiety, depression, and stress in Peruvian adults: a model of structural equations

**DOI:** 10.3389/fpsyt.2026.1862134

**Published:** 2026-08-17

**Authors:** Oscar Mamani-Benito, Maria Gracia Rodríguez-Díaz, Andy Sánchez-Villena, Victor Manuel Siesquen Custodio, Madeleine Huayta-Meza, Cristhian Cruz-Campos, Wilfredo Oscar Sucapuca Mamani, Rosa Farfán-Solís, Milagros Pacheco-Vizcarra, Josué E. Turpo-Chaparro

**Affiliations:** 1 Facultad de Ciencias de la Salud, Universidad Señor de Sipán, Chiclayo, Peru; 2 Dirección de investigación, Universidad Señor de Sipán, Chiclayo, Peru; 3 Escuela Profesional de Psicología, Facultad de Ciencias de la Salud, Universidad Peruana Unión, Juliaca, Peru; 4 Facultad de Ciencias de la Salud, Universidad Peruana Unión, Juliaca, Peru; 5 Universidad Continental, Lima, Peru; 6 Facultad de Enfermeria, Universidad Nacional del Altiplano, Puno, Peru; 7 Facultad de Ciencias Contables y Administrativas, Universidad Nacional del Altiplano, Puno, Peru; 8 Facultad de Ciencias Humanas y Educación, Universidad Peruana Unión, Lima, Peru

**Keywords:** anxiety, concern about victimization, depression, extortion, Peru, stress

## Abstract

**Introduction:**

Extortion has become a growing problem for public security in Latin America, especially in Peru, where an increase in the rate of victims of extortion has intensified citizens’ fear.

**Objective:**

The objective was to explore the relationship between worry about the possibility of being a victim of extortion and symptoms of depression, anxiety, and stress.

**Methods:**

A cross-sectional explanatory study was conducted with 1222 Peruvian adults (59.25% men; average age = 26.51 years) from the three regions of Peru (coast, mountains, and jungle), selected through intentional non-probabilistic sampling. Participants completed the Concern about Becoming a Victim of Extortion Scale (BECS; 8 items) and the Depression, Anxiety, and Stress Scale (DASS-13). With the data, a structural equation model with the WLSMV estimator was used to test the hypothetical model.

**Result:**

The structural model demonstrated an adequate adjustment (CFI = 0.986, TLI = 0.984, RMSEA = 0.067, SRMR = 0.036). In this case, worry about being a victim of extortion showed a large, direct, and significant effect on stress (β=0.553, R²=0.306) and anxiety (β=0.519, R²=0.269). At the same time, it had a moderate, direct, and significant effect on depression (β=0.427, R²=0.182).

**Conclusion:**

Concern about the possibility of being a victim of extortion is significantly associated with higher levels of stress, anxiety, and depression in the sample studied. These findings are preliminary and require replication in probabilistic samples before generalizing them to the Peruvian population.

## 1 Introduction

Extortion and other crimes have become one of the most relevant social problems (1, 2). These criminal manifestations not only put people’s physical and economic integrity at risk but also significantly impact their mental health (3, 4). In fact, prolonged exposure to environments of threat, violence, and uncertainty leads to the appearance of mental disorders such as depression (5), anxiety, and post-traumatic stress (6).

Under this scenario, the United Nations Security Council reports that violence linked to organized crime reached alarming levels worldwide (7), with 83% of the global population living in conditions of high crime (8). In Latin America, extortion represents a critical challenge of particular concern in countries such as El Salvador, Colombia, Honduras, and Peru (8), which has forced 1.2 million people to leave their homes due to crime (9).

In the Peruvian context, the most recent data reveal an alarming situation, since in the last months of 2024, 27.5% of the population over the age of 15 reported having been a victim of a crime (10). This insecurity has driven significant increases in crime, with a 3% increase in homicides (2,118 cases) and an 18% increase in extortion nationwide in recent years. This trend closed in 2025 with more than a 20% increase in extortion complaints compared to the last two years (11, 12). As a direct consequence, between 30% and 40% of cases of anxiety and stress are explicitly linked to experiences of theft, aggression, or constant perception of danger, underscoring the profound psychological impact of these crimes on collective mental health (6).

Beyond the figures, it is essential to understand the phenomenon from a psychological perspective, so it is important to distinguish between actual victimization by extortion and concern about the possibility of becoming a victim of this crime. While the first involves a direct experience of threat or coercion with documented economic and psychological consequences (13), the second refers to a psychological state of cognitive and emotional anticipation that can be present even in people who have never been direct victims of extortion (14). As such, extortion is understood as the imposition of payments under threat or intimidation (15, 16), while the concern about the possibility of becoming a victim is conceived as a multidimensional construct that encompasses four dimensions: cognitive (recurrent thoughts about the risk of victimization)17), emotional (anxiety, nervousness, and discomfort)18), behavioral (avoidant behaviors to minimize exposure to risk) (17) and perceived control (confidence to face situations)20).

This conceptualization is based on key theoretical models such as the (18, 19)fear of crime theory (20), rational choice theory, routine activity theory (21), and protection motivation theory (22). In the Peruvian context, where extortion rates have increased steadily (11, 12), the study of this anticipated concern is particularly relevant, since it affects a much broader segment of the population than that of direct victims.

On the other hand, the main mental health alterations associated with crimes such as extortion are depression, represented by the presence of low positive affect, along with sadness, hopelessness, and anhedonia; anxiety, which implies feeling tension accompanied by physiological agitation; and stress, which is a reaction to the continuous activation of daily demands that exceed the capacity to cope (23). This differentiation is supported by the tripartite model of Clark and Watson (24).

Here, it is important to conceptually distinguish extortion from other crimes such as theft, homicide, or organized crime. While robbery is usually a unique and short-lived event, and homicide involves lethal physical violence at a specific time, extortion is characterized by a continuous threat over time, a coercive relationship that can last for months or years, and the periodic demand for payments under intimidation (13). Unlike other property crimes, extortion establishes a link between victim and victimizer that generates a sustained state of alert and a chronic anticipation of damage (16, 25). This prolonged nature makes extortion a chronic stressor, while crimes such as theft operate as acute stressors (3). In addition, the concern about the possibility of becoming a victim of extortion activates mechanisms of anxious anticipation and avoidant behaviors that are not usually observed with the same intensity in the face of low-probability crimes or a single event (14). However, despite these differences, all these crimes share elements that affect mental health: exposure to threats, the perception of vulnerability, and the activation of stress responses (3).

Although the literature does not yet show direct evidence about the effect of worry about the possibility of being a victim of extortion on depression, anxiety, and stress, previous studies provide important information to understand how this problem, represented in crimes such as robbery, homicides, and organized crime, influences the emotional state of the general population. For example, in the United States, Weisburd et al. (26) found that residents of high-crime areas present 85% more symptoms of post-traumatic stress. Similarly, immigrant victims of extortion showed significant severity in post-traumatic stress (13). In Chile, Duarte et al. (27) showed that perceived insecurity and victimization by crimes elevated psychological distress (stress, depression, and general malaise).

Another group of studies accounts for the link between these crimes and anxiety. For example, in Brazil, Orellana et al. (28) demonstrated that victimization by theft is associated with a higher prevalence of generalized anxiety in adults. Likewise, Gonggrijp et al. (29) reported that victimization of property, violence, and sexual crimes is associated with higher levels of anxiety in Dutch adults. On the other hand, an analysis of textual data on sextortion showed that, in the short term, it generates concern, stress, and somatic symptoms of anxiety. In contrast, in the long term, it causes persistent episodes of anxiety (30). Likewise, Portuguese adolescents who were victims of polyvictimization presented higher levels of anxiety (31). Finally, Macassa et al. (32) found that Swiss women with a greater fear of being robbed reported more intense anxious symptoms.

Regarding the relationship with depression, this is also documented. For example, in Mexico, Balmori-de-la-Miyar et al. (5) found that victimization by violent crimes in adolescents increases depressive symptoms. Concurrently, Weisburd et al. (26) reported that residents of high-crime areas in the United States present 61% more depressive symptoms than those who live in areas with low crime. In addition, a systematic review identified that one of the most frequent psychological effects among crime victims is depressive symptoms (33). In Germany, Kilian et al. (34) showed that criminal victimization is significantly associated with depression. In another context, a population study in Sweden showed that adolescent victims of theft who did not report the crime to the police presented higher levels of psychological discomfort, including depressive symptoms, compared to those who did report it (35).

Consequently, it is possible to deduce that the concern about the possibility of being a victim of extortion has a significant impact on the mental health of the Peruvian population (6), since this is presented as a phenomenon aggravated by the rise in organized crime in the country. However, there is a gap in knowledge; despite the importance of the problem, there have been no studies that explain the effects on this specific population, since most focus on the experience of victims from criminological, political, or economic perspectives. Therefore, this research fills this gap by examining the factors that put the mental health of Peruvian citizens at risk. This will allow us to identify previously unexplored risk groups, design evidence-based prevention strategies, and inform public policies with effective interventions.

**Figure 1:**
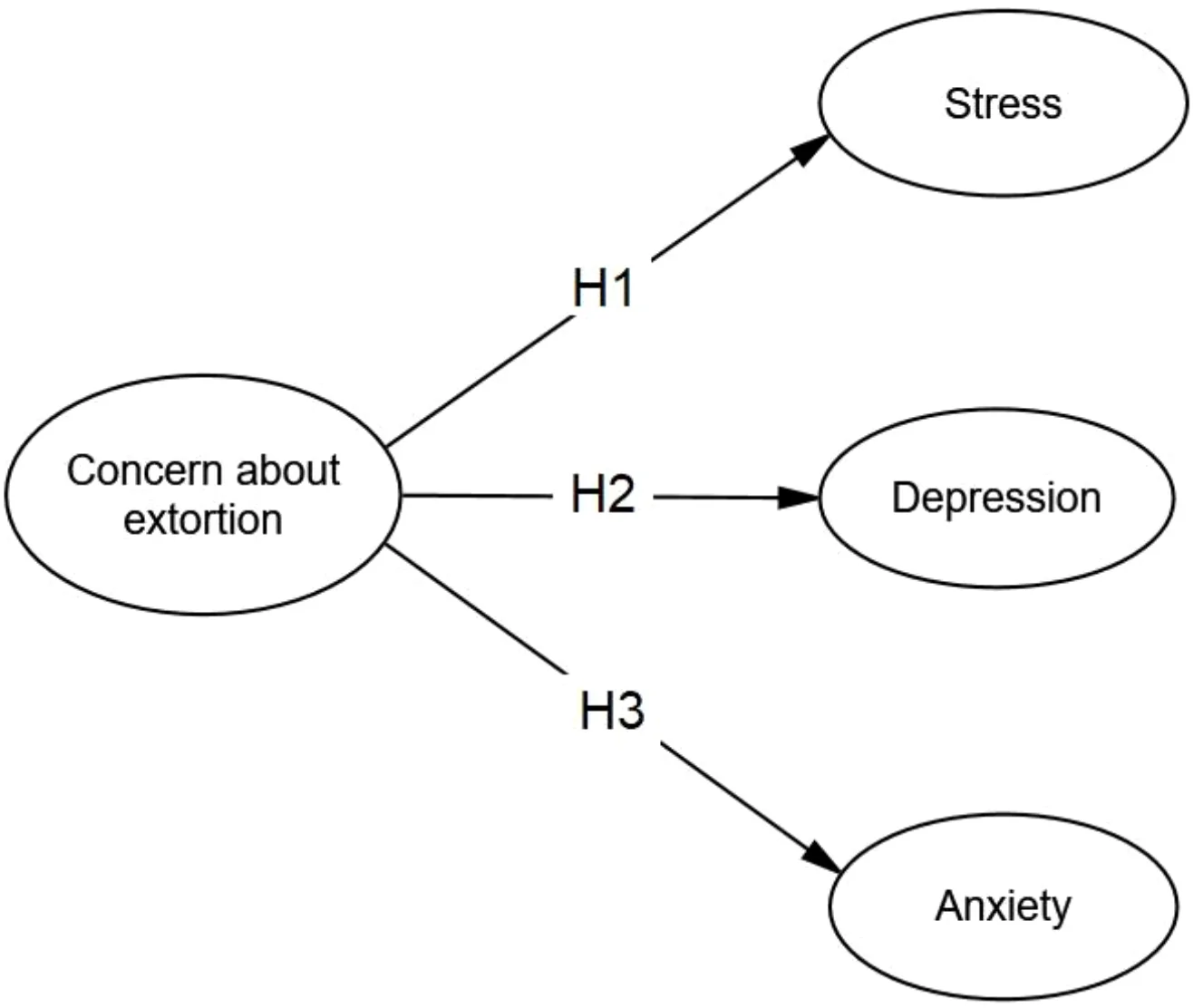
Hypothesized model.

Based on the arguments, the following study hypotheses are proposed (See **Figure 1**):

- H1: Concern about the possibility of being a victim of extortion is directly and significantly related to stress.
- H2: Concern about the possibility of being a victim of extortion is directly and significantly related to depression.
- H3: Concern about the possibility of being a victim of extortion is directly and significantly related to anxiety.

Therefore, the objective of this research was to explore the relationship between worry about the possibility of being a victim of extortion and symptoms of depression, anxiety, and stress in a sample of Peruvian adults.

## 2 Methods

### 2.1 Design

A cross-sectional explanatory study (36), since it is proposed to test a model about the relationships between a set of variables.

### 2.2 Participants

The *a priori* sample calculation was performed from the *a priori* statistical power approach using the semPower package in R. In the configuration, the parameters of α = 0.05, power = 0.80, and approximately 183 degrees of freedom were established because the initially hypothesized model contemplates four latent variables and 21 observable variables, and RMSEA as a measure of the effect = 0.05. Given this, the calculation estimated a minimum number of 117 participants.

The recruitment was carried out through intentional non-probabilistic sampling through social networks (mainly Facebook), taking into account the following inclusion criteria: (a) being over 18 years of age, (b) residing in Peruvian territory, (c) voluntarily accepting to participate through informed consent. No exclusion criteria were established related to mental health status (e.g., current diagnosis or treatment for anxiety or depression), or history of victimization for other crimes, or status as an active victim of extortion. Neither were the participants asked if they were receiving psychological or psychiatric treatment, nor if they had been previous victims of other crimes. These aspects are recognized as limitations of the study.

Under these conditions, the initial participation of 1325 volunteer participants was obtained. Of this number, 103 records were eliminated due to the omission of informed consent (n = 41), minority status (n = 53), and the presence of empty boxes (n = 9). Thus, the final sample consisted of 1222 Peruvian adults, with an average age of 26.51 years (SD = 9.11) of both sexes (59.25% men). More detailed sociodemographic characteristics can be seen in **Table 1**.

**Table 1:** Sociodemographic characteristics of the participants.

Variable	Category	n	%
Age		(M = 26.51)	(SD= ± 9.11)
Sex	Male	724	59.25
	Female	498	40.75
Education	University	923	75.53
	Secondary	277	22.67
	Elementary	22	1.80
Marital Status	Single	896	73.32
	Married or cohabitant	276	22.59
	Divorced	35	2.86
	Widow (er)	15	1.23
Region	Mountain	592	48.45
	Coast	371	30.36
	Jungle	259	21.19
Type of work	Dependent (Salaried)	673	55.07
	Independent (entrepreneur, trader, businessman)	549	44.93

*f*, frequency; %, Percentage.

To evaluate the representativeness of this group, its characteristics were compared with the preliminary results of the National Census reported by the National Institute of Statistics and Informatics (37). This analysis revealed significant discrepancies from the general population. First, the sample presented a marked overrepresentation of males (59.25%) in contrast to the national census distribution (49.4%). Second, a bias towards a significantly younger age profile was found, registering a median age of approximately 23 years compared to the national median of 32 years. Finally, the distribution by natural regions showed an underrepresentation of coastal residents (30.36%) compared to 57% reported in the census, an overrepresentation of the mountains (48.45%) compared to 29% of the census, as well as 21.19% of the jungle compared to 13% reported in the census. These divergences confirm that the sample is not statistically representative of the general Peruvian population; therefore, the findings are strictly limited to the segment evaluated and should not be extrapolated at the national level without due caution.

### 2.3 Instruments

Concern about Becoming a Victim of Extortion Scale (BECS): It is a self-report instrument developed in the Peruvian context (14) and has 8 items that are grouped into a single dimension. The response scale is Likert-type with 5 points ranging from Never = 1 to Always = 5. The instrument has evidence of content validity in three criteria (clarity, relevance, and representativeness), as well as internal structure validity demonstrated with confirmatory factor analysis (SRMR = 0.037, RMSEA = 0.074, CFI = 0.996, TLI = 0.994) and convergent validity with scales that measure concern about being a victim of theft (r = 0.74) and anxiety (r = 0.36). In addition, it demonstrated invariance according to sex and reliability by internal consistency (ω > 0.90). The internal structure was also demonstrated with the data of this study through confirmatory factor analysis, whose results indicated a one-dimensional structure with good fit and good reliability (CFI = 0.980, TLI = 0.972, RMSEA = 0.178 [90% CI 0.168 - 0.189], SRMR = 0.037, ω = 0.957), in addition, its factorial loadings were high, with λ > 0.80 values in all dimensions.

Depression, Anxiety, and Stress Scale (DASS): It is a self-report instrument that has 13 items in its abbreviated version, which was validated in Peru (38). The DASS-13 has four response options ranging from Not at all = 1 to Much/Most of the time = 4. It is grouped into three dimensions called depression, anxiety, and stress (CFI = 0.961; TLI = 0.951; RMSEA = 0.076; SRMR = 0.028), although it was also shown to have a bifactor structure (CFI = 0.988; TLI = 0.983; RMSEA = 0.059; SRMR = .026) with a high reliability for the overall factor (H = 0.94). In this study, the internal structure was demonstrated with confirmatory factor analysis, testing the three-dimensional model (CFI = 0.996, TLI = 0.995, RMSEA = 0.056 [CI 90% 0.049 - 0.062], SRMR = 0.016, ω_stress_ = 0.905, ω_anxiety_ = 0.901, ω_depression_ = 0.924; factorial loads λ > 0.80 in all dimensions), bifactor (fit indices could not be estimated), second-order (CFI = 0.996, TLI = 0.995, RMSEA = 0.056 [CI 90% 0.049 - 0.062], SRMR = 0.016, ω_stress_ = 0.905, ω_anxiety_ = 0.901, ω_depression_ = 0.924; factorial loads λ > 0.80 in all specific dimensions) and one-dimensional (CFI = 0.985, TLI = 0.982, RMSEA = 0.104 [CI 90% -0.98 - 0.110], SRMR = 0.032, ω = 0.971; λ factorial loads > 0.80 in all dimensions).

### 2.4 Procedure

The data were collected virtually. Therefore, an online form was designed, which was distributed through social networks, mainly Facebook, through a direct access link. In this case, this resource was chosen for several reasons (39), for example, the speed and efficiency that reduces the time and effort in the management and processing of information, the reduction of human errors associated with manual transcription and coding, the accessibility and scope that allows participants located in different geographical regions, the flexibility for participants to respond at the time and place of their choice, and finally, for the lowest costs, since printing, travel, and personnel expenses are eliminated when applying the survey.

The resource created in Google Forms contains the following structure: informed consent, a section where the purpose of the study is explained, highlighting voluntary and anonymous participation, and finally, an instrument items section. The estimated time to complete this form was 5 to 10 minutes. To avoid duplicate responses, the Google Form was configured to allow a single response per user, requiring login with an email account. Additionally, the timestamps of the responses were reviewed to identify possible duplicates.

### 2.5 Data analysis

The data analysis was carried out at various times and was carried out with R 4.4.3. software and the lavaan and dplyr packages. In the first step, data cleaning was carried out, for which 103 responses were eliminated because they were minors (n = 53), others did not agree to be part of the study, as they rejected informed consent (n = 41), and for empty boxes (n = 9). Then, a descriptive analysis of the data was performed, using the mean, standard deviation, kurtosis, and skewness, as well as the response rate for each option. Regarding kurtosis and asymmetry, a cut-off point of ±1.5 was taken into account (40). In addition, a correlation matrix was obtained, expecting p-values <.05.

Next, the measurement models of the instruments were tested in order to have an adequate structural model, based on the internal structure of the DASS and BECS. To do this, we began by testing 4 factorial models of the DASS-13: the first was the three-dimensional one with the intercorrelated factors of depression, anxiety, and stress; the second was a bifactor model with a general factor and three specific orthogonal ones due to the high correlation between the Φ >.89 dimensions (41); the third was a second-order model with a general factor that explains the three specific ones. This decision was made because the bifactor model absorbed almost all the common variance of the items, generating little variance of its own in the dimensions, which generated unstable parameters and negative variances that hinder the interpretation of the model; in fact, the anxiety dimension disappears, and item 9 has an extreme load. In the fourth model, a one-dimensional model was evaluated with the 13 items of the DASS-13. The analysis of the measurement model was also carried out with the BECS, assuming a one-dimensional structure. To identify the adjustments, the CFI > 0.90, TLI > 0.90, RMSEA < 0.08, or that the upper limit of the confidence interval is less than.100, and SRMR < 0.08 were used (42, 43). However, priority was given to the SRMR because the RMSEA usually penalizes simple models with few degrees of freedom, which occurs in instruments with few items (44), before which more flexible cut-off points have been proposed, such as < 0.200 for RMSEA when there are factorial loads close to 0.90 (45). The estimates were made with WLSMV for the ordinality of the items. In addition, the reliability was calculated with the ordinal omega coefficient, expecting values greater than 0.70 (46).

Next, the structural model was created with structural regression, also using WLSMV as an estimator, given the ordinal nature of the data (47). At this point, 3 different models were tested, depending on the measurement models of the DASS-13. The first assumes that the concern about the possibility of being a victim of extortion explains depression, anxiety, and stress, which are correlated (M1); the second, the concern about the possibility of being a victim of extortion explains a general factor of discomfort that in turn explains depression, anxiety, and stress, which are specific factors and that corresponds to the second-order model (M2); the third and last, is similar to M2 but with the difference that the concern about the possibility of being a victim of extortion also explains the specific factors (M3). Finally, the CFI > 0.90, TLI > 0.90, RMSEA < 0.08, and SRMR < 0.08 indices were used to evaluate the fit (42, 43), and the proposed models were graphed with the semPlot package.

Finally, the structural invariance was verified according to sex (men vs. women), region (coast vs. mountain vs. jungle), and type of work (dependent vs. independent), for which a base model was created to which it was compared with a model restricting the latent regression coefficients to see later if there are differences in the groups. For this, the change in the CFI and the RMSEA will be taken into account, expecting ΔCFI > 0.01 and ΔRMSEA < 0.015 to conclude invariance in the groups (48). In addition, the base and restricted models will be compared by waiting for a p-value > 0.05 to affirm that there are no differences and, therefore, the invariance is met (49).

In addition, for this research, the influence was also analyzed from an effect magnitude approach: an R^2^ less than 0.02 was considered insignificant, between 0.02 and 0.13, small; between 0.13 and 0.26 moderate, and greater than 0.26, large (50).

Both the raw database and the R codes are available at: https://osf.io/jfp7q/overview.

### 2.6 Ethical considerations

This research was conducted in strict compliance with the Declaration of Helsinki (51). In addition, it has the approval of the ethics committee of the Señor de Sipán University (Code: 1573-CIEI).

## 3 Results

**Table 2** shows that the highest mean is the concern about the possibility of being a victim of extortion (M = 23.176; SD = 8.187), while the lowest mean is anxiety (M = 8.238; SD = 3.180). Regarding normality, all variables have values below 1.5, concluding that there is univariate normality. Regarding the correlation matrix, concern is more closely linked to stress (r = 0.495).

**Table 2:** Descriptive statistics.

Variable	Mean	SD	Skewness	Kurtosis	Concern	Stress	Anxiety	Depression
Concern	23.176	8.187	0.122	-0.928	1	0.495***	0.461***	0.379***
Stress	10.525	3.718	0.438	-0.548	0.495	1	0.863***	0.804***
Anxiety	8.238	3.180	0.480	-0.610	0.461	0.863***	1	0.847***
Depression	7.877	3.335	0.560	-0.671	0.379	0.804***	0.847***	1

p < 0.05, **p < 0.01, ***p < 0.001.

As for the structural models, **Table 3** shows that all of them have acceptable adjustments, especially M1 (CFI = 0.986, TLI = 0.984, RMSEA = 0.067 [0.063 - 0.071], SRMR = 0.036) and M2 (CFI = 0.984, TLI = 0.982, RMSEA = 0.072 [0.068 - 0.071], SRMR = 0.042), since M4 showed an RMSEA slightly above the suggested cut-off point (CFI = 0.978, TLI = 0.976, RMSEA = 0.084 [0.080 - 0.087], SRMR = 0.048) and M3 could not estimate the fit indices.

**Table 3:** Goodness of fit indices of structural models.

Model	CFI	TLI	RMSEA [90% CI]	SRMR
M1	0.986	0.984	0.067 [0.063 - 0.071]	0.036
M2	0.984	0.982	0.072 [0.068 - 0.071]	0.042
M3	–	–	–	–
M4	0.978	0.976	0.084 [0.080 - 0.087]	0.048

**Figure 2:**
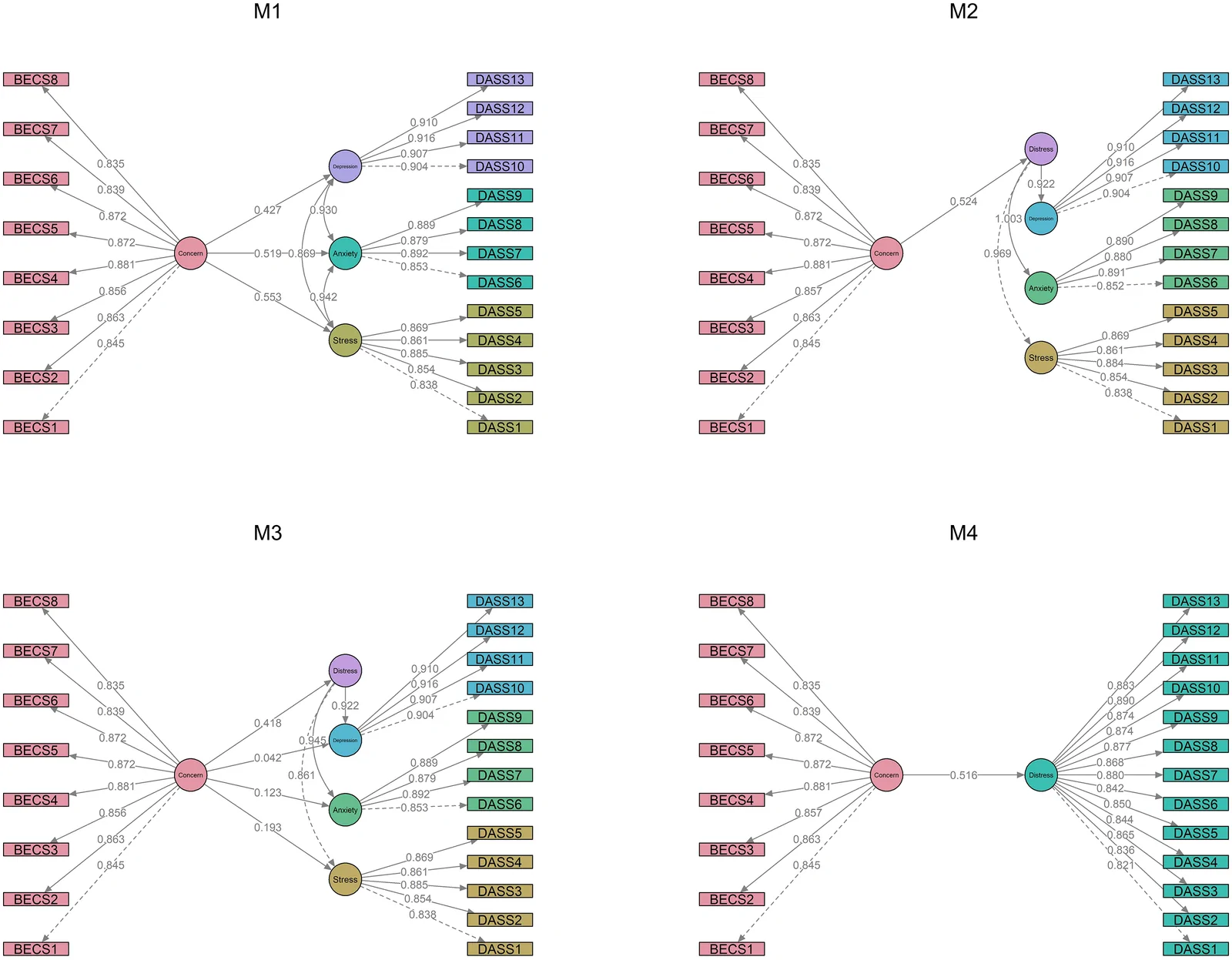
Structural regression models.

**Figure 2** shows the graphical representations of the four models tested. In M1, it is observed that worry about the possibility of being a victim of extortion has a direct and large effect on stress (β = 0.553; R^2^ = 0.306, p = 0.000); a direct and large effect on anxiety (β = 0.519; R^2^ = 0.269, p = 0.000) and a direct and moderate effect on depression (β = 0.427; R^2^ = 0.182, p = 0.000). In M2, it is shown that worry about being a victim of extortion has a direct and large effect on the hierarchical factor called psychological distress (β = 0.524; R^2^ = 0.274, p = 0.000), which is composed of/predicts depression (β = 0.922; R^2^ = 0.850, p = 0.000), stress (β = 0.969; R^2^ = 0.939, p = 0.000), and anxiety (β = 1.003; R^2^ = NA, p = 0.000); in the latter, a marginal Heywood case is shown by the slight negative variance, extremely close to zero, and not statistically significant (θ = -0.004, p = 0.507), which may be due to high collinearity (52, 53).

On the other hand, in M3 it is shown that worry about the possibility of being a victim of extortion has a direct and large effect on a hierarchical factor called psychological distress (β = 0.418; R^2^ = 0.175, p = NA) and on the specific factors depression (β = 0.042; R^2^ = 0.002, p = NA), anxiety (β = 0.123; R^2^ = 0.015, p = NA) and stress (β = 0.193; R^2^ = 0.037, p = NA); however, standard errors and statistical significance could not be estimated, which may be a symptom of poor model specification. Finally, in M4, it is observed that worry about the possibility of being a victim of extortion has a direct and large effect on psychological distress in general (β = 0.516; R^2^ = 0.267, p = 0.000).

After these results, it was decided to choose M1 for three reasons: the first, because it is the model with the best fit compared to the other alternatives; the second, because the measurement model of the instrument that evaluates depression, anxiety, and stress, showed an exactly equivalent fit between the three-dimensional structure and the second-order one, however, under the principle of parsimony, it is more appropriate to opt for the simplest model; the third because although the hierarchical model is also adequate even in other studies, the structure of the DASS is still the subject of debate in the literature, particularly regarding whether it measures a general construct of emotional discomfort or three specific ones, since the high correlation may indicate overlap in the factors for comorbidity, but at a theoretical and conceptual level, depression, anxiety, and stress are conceptually differentiated constructs (38). Therefore, it was considered more prudent to keep the three-factor solution proposed in M1.

Regarding the invariance of the model, **Table 4** shows that the structural invariance of M1 is met for each subgroup, since the differences between the models were not statistically significant and, in addition, the values of ΔCFI and ΔRMSEA improve in the restricted model. Therefore, it is concluded that sex, region, and type of work are not variables that interact/moderate the relationships between worry about the possibility of being a victim of extortion and depression, anxiety, or stress.

**Table 4:** Structural invariance according to sex, region, and type of work.

Group	Model	CFI	RMSEA	χ² diff (df)	p-value	ΔCFI	ΔRMSEA
Sex	Base	0.985	0.070				
	Restricted	0.986	0.068	5.555 (3)	0.135	0.001	-0.002
Region	Base	0.985	0.070				
	Restricted	0.987	0.067	7.257 (6)	0.298	0.001	-0.003
Type of work	Base	0.986	0.068				
	Restricted	0.987	0.065	2.342 (3)	0.505	0.001	-0.003

**Figure 3:**
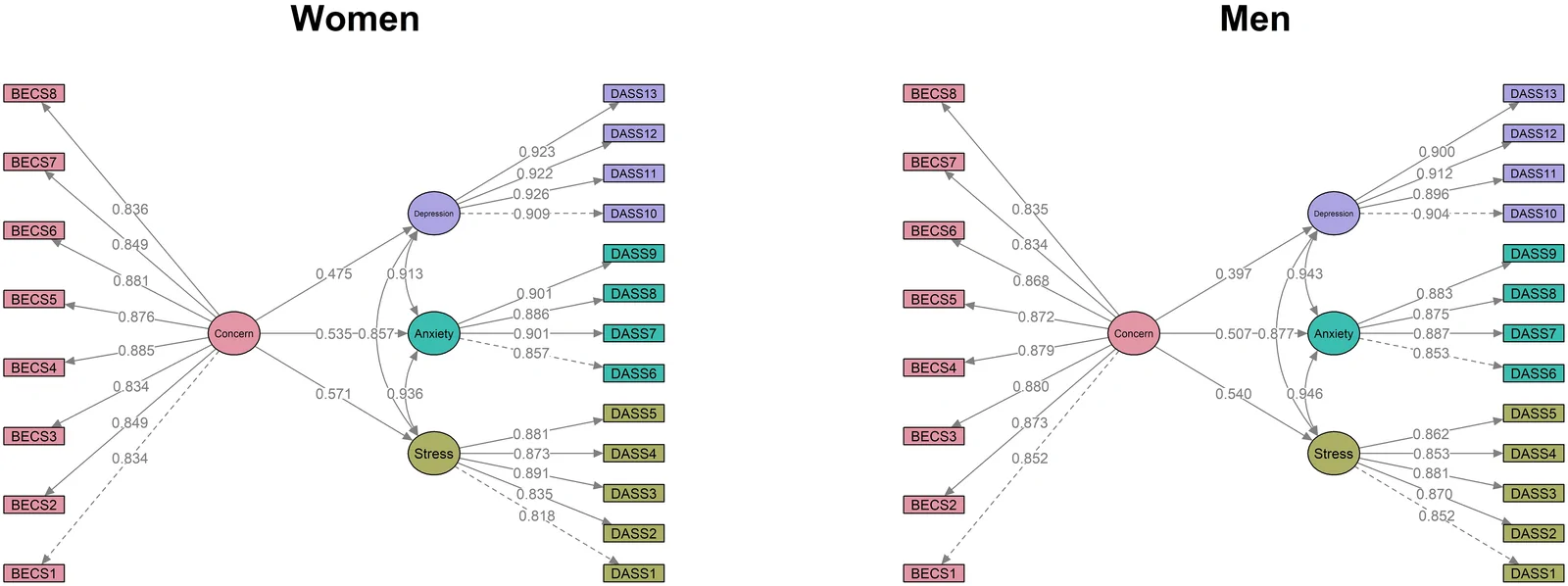
Structural regression models according to sex.

Despite the invariance, at the descriptive level, there are certain differences in the effects of worry about the possibility of being a victim of extortion on depression, anxiety, and stress according to sex, since women present higher values than men (See **Figure 3**).

**Figure 4:**
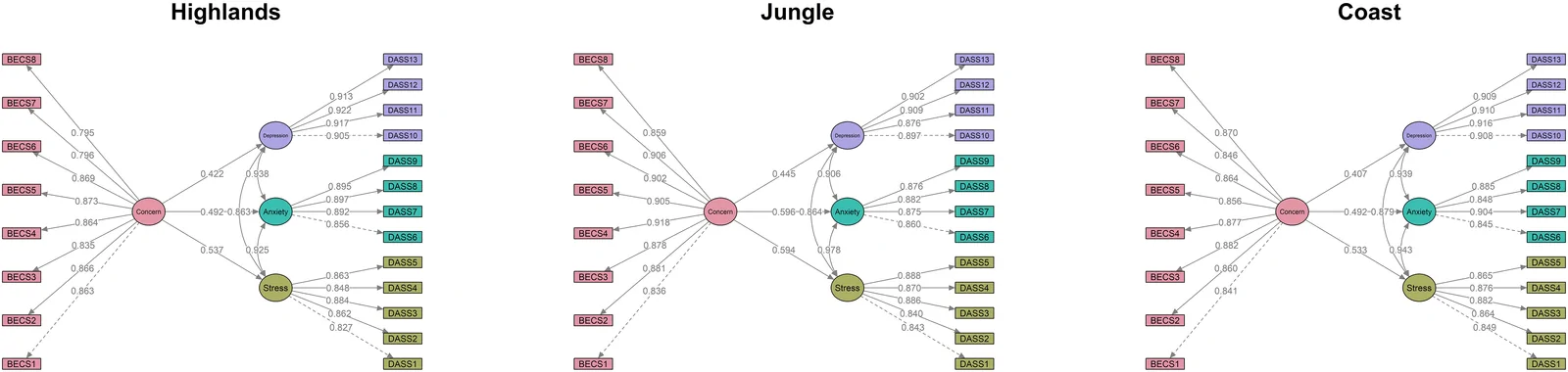
Structural regression models according to region.

**Figure 4** also shows differences in the effects of worry about the possibility of being a victim of extortion, since the inhabitants of the jungle have higher values, followed by those who live in the mountains. In contrast, those on the coast have fewer effects.

**Figure 5:**
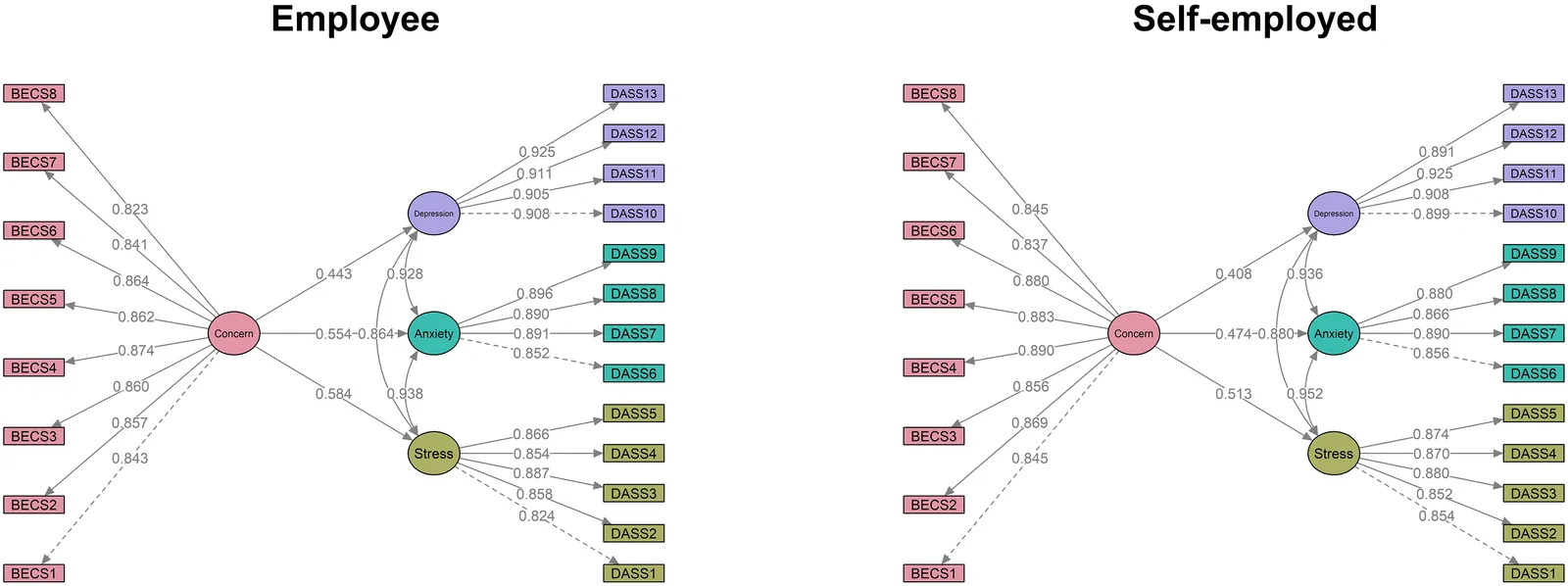
Structural regression models according to type of work.

Finally, when comparing the effects according to the type of work, dependent workers are those who presented greater values in the affectation of worry about the possibility of being a victim of extortion, depression, anxiety, and stress (See **Figure 5**). On the other hand, in all subgroups, it is observed that worry predicts, to a greater extent, anxiety and stress.

## 4 Discussion

Extortion, along with economic threats, is closely related to increased symptoms of anxiety and depression (13). In addition, the experience of extortion can be a trigger for the development of post-traumatic stress, especially in contexts where people are exposed to different forms of violence and coercion (25). On the other hand, victims of extortion may experience panic, fear, anxiety, and depression, and different dynamics of coercion (14). In this sense, the objective of the present research was to explore the relationship between concern about the possibility of being a victim of extortion and the symptoms of depression, anxiety, and stress in a sample of Peruvian adults.

A first relevant finding shows that the M1 structural model is the most appropriate model to understand the relationship between the variables. Although alternative specifications were evaluated (M2, M3, M4), M1 showed a better overall fit and allowed the conceptual difference between stress, anxiety, and depression to be preserved. In addition, the dimensions of the DASS-13 were highly correlated, but preserving the conceptual difference between stress, anxiety, and depression (23). This means that worry about the possibility of being a victim of extortion not only increases general emotional distress but is expressed in a differentiated way in responses to stress, anxiety, and depression, which is articulated with the tripartite model of Clark and Watson (24).

The literature has reported that crime acts as a chronic stressor that generates fear and uncertainty through direct victimization or indirect exposure, which is associated with psychological distress, including anxiety and depression (3). In this sense, constant concern about the possibility of suffering extortion can activate stress mechanisms (54). From the theory of protection motivation, vulnerability to threat can activate motivational and psychological responses to risk (22).

The M1 also showed that the highest observed effect was on stress, which means that extortion represents an anticipated threat that often leads to intimidation, coercion, and a perception of vulnerability (16). It is likely that the person who has not yet been a direct victim, however, due to the possibility of being a victim, can be kept in a continuous state of alert (3). The literature shows that threatening experiences can impair mental well-being and increase stress responses (25). Along the same lines, the second strongest effect was on anxiety, indicating that concern about extortion not only generates general tension but also an anxious anticipation in the face of the possibility of harm. Concern about being a victim can generate recurrent thoughts of threat, hypervigilance, and avoidant behaviors that can increase anxiety (28).

For Peruvian individuals, the concern about the possibility of being a victim of extortion involves emotional and cognitive processes, since uncertainty increases anxiety (55). This worry aggravates stress, creating a cycle of anxiety that hinders recovery (56). In this sense, the literature confirms that concern about the possibility of being a victim of extortion is associated with a greater likelihood of reporting anxiety (32).

Regarding depression, a direct and significant effect was reported, although of lesser magnitude than that observed for stress and anxiety. This means that worry about extortion activates alert and tension mechanisms closer to states of stress and anxiety. However, when that threat is perceived as a loss of security, it could favor feelings of depression. In this regard, previous studies show that, for example, exposure to violent crimes increases depressive symptoms (5) and that prior concern about being a possible victim is linked to depressive symptoms (34). On the other hand, the effect of lesser magnitude on depression should be interpreted moderately, since depression could require longer processes of loss of resources or deterioration of social support to manifest itself with greater intensity. Instead, stress and anxiety appear more immediately in the face of the threat.

It is important to highlight the high association between stress, anxiety, and depression, which supports the presence of a common component of emotional distress, as noted by Peña-Tomas et al. (38). However, the chosen model allows us to argue that these symptoms, although related, retain conceptual differences. Therefore, the results should not be interpreted as three completely independent processes, nor as a single answer. The result indicates that the concern about the possibility of being a victim of extortion activates a general system of discomfort that is expressed with distinction in the individual’s stress, anxiety, and depression.

Another contribution of the study is found in the analysis of invariance according to sex, region, and type of work. The results showed that the model works equally in men and women, in participants from the coast, the mountains, and the jungle, and in dependent and independent workers. This finding confirms the consistency of the model, as it suggests that concern about the possibility of being a victim of extortion shows similar relationships in different subgroups of the sample (by sex, region, and type of work), suggesting that the pattern of association is consistent internally. However, its generalization to the Peruvian population requires confirmation with probabilistic samples. In this case, from the framework of Cohen and Felson (21), the concern about the possibility of being a victim of extortion functions as an activator of vigilance and tension; and from the perspective of Gabriel and Greve (19), that concern triggers cognitive and emotional processes that can be understood as stress, anxious activation, and a low effect typical of depression.

Among the practical implications is the need to establish preventive interventions with the aim of helping citizens manage and cope with the concern of being victims of crime in order to improve mental health. Likewise, municipalities and government agencies must implement community fear management programs along with practical and real crime control policies. It is also necessary to carry out mass communication campaigns that show the route of action in the face of a threat of extortion.

Despite the interesting results, this research has methodological limitations that must be considered for the correct interpretation of the findings. First, the study design was cross-sectional, which prevents establishing causal relationships or definitive temporal directionality between concern about extortion and psychological symptomatology; therefore, the models presented must be interpreted strictly in terms of structural associations. Secondly, although the sample size is robust (n = 1222), non-probabilistic online recruitment generated representativeness biases against the national demographics, evidenced by a marked concentration of young participants, males, and mountain residents. This method also introduces biases of self-selection and volunteering, since the people who decided to participate may systematically differ from those who did not. Together, these limitations restrict the generalization of the results to the entire Peruvian population, so future research should employ probabilistic or stratified sampling to balance territorial and demographic representativeness.

Third, data collection relied exclusively on self-reporting instruments managed in virtual environments. This modality introduces the possibility of common response biases, such as social desirability or the under/overrepresentation of affective symptoms in the absence of a face-to-face clinical evaluation. Likewise, given the multifactorial nature of the phenomenon, the model did not contemplate critical contextual variables such as the previous experience of direct or indirect victimization (personal or family), a factor that the literature reports as a powerful catalyst of the perception of threat and the consequent distress. Fourth, although the analysis of invariance suggests that the structural model operates equivalently between the groups, at the descriptive level, higher coefficients of association were observed in women, forest residents, and dependent workers. Although these variations do not constitute formal evidence of statistical moderation under the applied criteria, they do constitute a priority line of research. These patterns suggest the existence of specific vulnerabilities linked to labor dynamics of subordination, factors of territorial isolation, or gender gaps in the perception of citizen insecurity that deserve to be examined in depth in future explanatory studies.

Finally, no information was collected on whether the participants were receiving treatment for any mental health disorder, if they had been previous victims of other crimes, or if they were active victims of extortion at the time of the study. These factors could influence the levels of concern and symptoms reported. Therefore, future research should control for these variables to isolate the specific effect of extortion concern more accurately.

## 5 Conclusion

Despite these limitations, we consider this research a solid contribution to the literature on the effect of worry about the possibility of being a victim of extortion on mental health. Therefore, we conclude that concern about the possibility of being a victim of extortion is significantly associated with higher levels of stress, anxiety, and depression in the sample of Peruvian adults studied. However, due to the cross-sectional design and non-probability sampling, these findings are preliminary. Studies with probabilistic samples and longitudinal designs in different Latin American contexts are required to confirm the directionality and generalization of these associations.

## Data Availability

The raw data supporting the conclusions of this article will be made available by the authors, without undue reservation.
